# The Association between Pelvic Asymmetry and Lateral Abdominal Muscle Activity in a Healthy Population

**DOI:** 10.5114/jhk/191098

**Published:** 2024-12-19

**Authors:** Maciej Biały, Wacław M. Adamczyk, Tomasz Stranc, Anna Gogola, Rafał Gnat

**Affiliations:** 1 Institute of Physiotherapy and Health Sciences, Jerzy Kukuczka Academy of Physical Education in Katowice, Katowice, Poland.; 2 Functional Diagnostics Laboratory, Sport-Klinika, Scanmed Sport, Żory, Poland.; 3 Laboratory of Pain Research, Institute of Physiotherapy and Health Sciences, Jerzy Kukuczka Academy of Physical Education in Katowice, Katowice, Poland.; 4 Piast Gliwice Football Club, Gliwice, Poland.; 5 Motion Analysis Laboratory, Institute of Physiotherapy and Health Sciences, Jerzy Kukuczka Academy of Physical Education in Katowice, Katowice, Poland.

**Keywords:** asymmetry index, postural activity, ultrasonography, lumbopelvic region, tissue deformation index

## Abstract

The human pelvis is subjected to forces generated by abdominal muscles. Pelvic asymmetry (PA) might therefore be related to the asymmetrical postural activity of the lateral abdominal muscles (LAMs: transversus abdominis (TrA); internal oblique (IO); external oblique (EO)). The main aim of this study was to evaluate the potential relationship between PA, expressed by the asymmetry index in the frontal (PAIf) and sagittal (PAIs) planes and LAM postural activity as described by the tissue deformation index (TDI). A group of 126 healthy volunteers (59 females) was involved. Positions of the anatomic landmarks for PA measurement were registered by the motion capture system. The response of LAMs to postural disturbation was recorded using the M-mode ultrasounds. We found weak negative correlations between PAIf and TDI values as well for the right and the left side of the body except for EO muscle (PAIf-TRA right: r = −0.11, left: r = −0.06; PAIf-IO right: r = −0.15, left: r = −0.10; PAIf-EO right: r = 0.02, left: r = 0.12). On the contrary, analysis between PAIs and TDI values revealed weak positive correlations, also except for EO muscle (PAIf-TRA right: r = 0.004, left: r = 0.003; PAIf-IO right: r = 0.05, left: r = 0.06; PAIf-EO right: r = 0.07, left: r = −0.02). For all tested correlations, we recorded non-significant outcomes (all p > 0.05). We found no evidence to support the claim that PA is related to the LAM activity in the group of young, healthy, and active people.

## Introduction

For several decades, clinicians have suggested that abdominal muscles and the pelvis compose one biomechanical unit, which is responsible for trunk control and for the ability to execute multiple functional tasks (Gnat and Saulicz, 2008; Gnat et al., 2009; Kim et al., 2006; Levine et al., 1997). In this specific musculoskeletal complex, the pelvis is continuously subjected to forces generated by abdominal musculature. Pelvic asymmetry (PA), which is a frequently seen phenomenon (Gnat and Biały, 2015), might therefore be related to the asymmetrical postural performance of the specific abdominal muscles. Interestingly, the occurrence of the two phenomena is common in both symptomatic (Ihlebæk and Lærum, 2004; Massé-Alarie et al., 2016) as well as asymptomatic populations (Krawiec et al., 2003; Marshall and Murphy, 2006).

Pelvic asymmetry refers to the asymmetrical alignment of the innominate bones. It is usually divided into two types: frontal PA (lateral pelvic tilt) and sagittal PA (iliac anterior/posterior rotation asymmetry) (Gnat and Biały, 2015). In some cases, PA is present in people with symptoms of low back pain (LBP) (Burton et al., 1995; Ihlebæk and Lærum, 2004) and is related to the movement asymmetry of the lower spine (Al-Eisa et al., 2006). Therefore, PA might be perceived as an essential factor contributing to lumbopelvic disorders (Yu et al., 2020). Al-Eisa et al. (2006) investigated the asymmetry of trunk movements in a sitting and a standing position in 54 people showing PA plus LBP and 59 asymptomatic subjects. They found that movement asymmetry was strongly associated with PA in both study groups, regardless of the position of the subjects’ body. Moreover, other authors claim that unilateral sport activities characterized by intensive use of one side of the body with frequent flexion and rotation of the trunk may also promote PA in a healthy population (Bussey, 2010; Cadens et al., 2023). In the study conducted by Krawiec et al. (2003) on 44 healthy athletes, PA was found in 95% of the subjects. The abovementioned data support findings presented by Gnat et al. (2009) who reported that asymmetric mechanical loads applied to the lumbopelvic complex could immediately cause PA in asymptomatic subjects, which was not the case after exposition to symmetrical loads.

Several studies have already demonstrated relationships between biomechanical properties of the sacroiliac joint (SIJ) and performance of specific abdominal and pelvic muscles. The effect of the transversus abdominis (TrA) and pelvic floor muscle activity on SIJ stiffness has been reported in both *in vitro* (Gnat et al., 2015) and *in vivo* settings (Richardson et al., 2002; Snijders et al., 1995). Snijders et al. (1995) noticed that TrA could increase compression forces in the sacroiliac joints. Richardson et al. (2002) assessed the effects of abdominal muscle activation on SIJ stiffness, and their results confirmed the dominant influence of TrA on the stiffness of the SIJ. The influence of the TrA on SIJ stiffness during locomotion-resembling movements of the innominate bones has also been tested in the *in vitro* setting by Gnat et al. (2015) with the contrary results. Those authors found that force generated by the TrA was not likely to increase stiffness in the SIJ. It is worth investigating whether such a relationship might adapt the opposite direction, i.e., from original asymmetrical arrangement of the two pelvic bones (due to, e.g., leg length discrepancy) and therefore the SIJs, to the modifications of the abdominal/pelvic muscles performance.

The activity of the muscular system of the lumbo-pelvic region may constitute a critical factor that can influence arrangement of the pelvic bones. The present study aimed to identify the relationship between PA and variables describing postural activity of lateral abdominal muscles (LAMs) in their response to the experimentally induced postural disturbation.

## Methods

### Participants

One hundred and fifty volunteers agreed to participate in the study. One hundred and twenty-six (59 females) were qualified (mean age: 22.87 ± 2.61 years; body mass: 69.89 ± 12.62 kg; body height: 174.07 ± 10.24 cm; body mass index: 22.90 ± 2.46 kg/m^2^). The inclusion criteria were: ≤ thirty years of age, male or female, stable level of physical activity (regular participation in physical activities with the minimum of 6 h per week, without any sudden changes (new sport, significantly increased/decreased volume of training, etc. within one month prior to the measurements)). The exclusion criteria were: history of serious pain or injury within the lumbopelvic area and lower extremities (requiring medical assistance for at least two weeks), history of any surgical intervention, pain or functional limitations within the upper extremities, no specific fitness activity engaging the abdominal musculature (Pilates, yoga, etc.) at least two months prior to the study, pregnancy, childbirth during one year before the study, pain or other minor inconveniences (e.g., cold, headache) on the day of measurement. All participants signed written informed consent. The research was approved by the Ethics Committee of the Jerzy Kukuczka Academy of Physical Education in Katowice, Katowice, Poland (approval code: 18/2007; approval date: 31 May 2007). All measurements were conducted in the laboratory setting under standardized environmental conditions.

### Raters

Two certified physiotherapists, each with two years of professional experience, were recruited as raters. Both were blinded to the objective of the study. They underwent four-week training in palpation of the anterior (AS) and posterior (PS) superior iliac spines. They also got familiar with the position capture system protocol for PA and the ultrasound (US) measurement protocol for LAM activation. Training was guided by an experienced specialist who was not directly involved in the study. Five tasks were randomly assigned to the raters and remained unchanged throughout the study: (1) palpation of the AS; (2) palpation of the PS; (3) operating the US array; (4) recording the US images; and (5) data readout from the recorded US images. A third researcher was also involved and was responsible for the procedure supervision, subjects enrolment, and data processing.

### Pelvic Asymmetry Measurement

**Figure 1:**
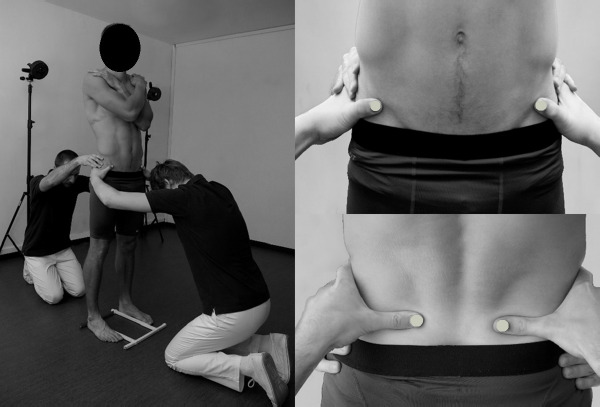
One of the participants and raters during the pelvic asymmetry measurement procedure. Reflective markers are attached to the rater’s thumbs and located on the anterior superior and posterior superior iliac spines.

To evaluate PA, the motion capture system BTS Smart (BTS Bioengineering, Milan, Italy) was used in a non-standard manner. In this system, reflexive markers placed on the surface of the subject's body are illuminated by the infrared light source, and reflections are captured by a set of six cameras. After computer processing, the *x, y*, and *z* coordinates of the markers in the given reference space are calculated with the frequency of 60 Hz. In our non-standard approach, raters' thumbnails were specially equipped with semispherical markers (diameter of 10 mm) for the purpose of iliac spines palpation and registration of their positions. To standardize foot placement, a plastic frame was mounted on the floor. Participants pushed the inner and posterior borders of their feet against its bars (the distance between the inner borders of the frame was 23.5 cm) (Gnat and Biały, 2015). After proper positioning of the participant, raters firmly grasped his/her pelvis placing thumbs equipped with markers directly under the target anatomical landmark, so that the upper border of their thumbs 'supported' either the ASs (rater 1) or PSs (rater 2). Subsequently, raters gave command 'start' to the technician operating the motion capture system. Three 5-second static registrations of the pelvic spine position, ASs and PSs, were recorded. This measurement method reduced the risk of marker detachment and the firm grasp of the pelvis by the two raters stabilized it and minimized the influence of the postural sway on the outcome. The reliability and the detailed methodology of the measurement have been reported earlier (Gnat and Biały, 2015) (Figure 1).

### Postural Activity of the LAMs

In the next step, postural activity of the LAMs was assessed using the Mindray DP 6600 US imaging device (Mindray, Shenzhen, China) equipped with a linear array and the sampling frequency of 5 MHz. In order to localize the individual LAMs, the US B-mode was engaged. Then, the device was switched to the M-mode, in which the final images were recorded.

Of our interest was the LAM response to postural perturbation in the form of active, rapid arm movement—abduction with the weight of 3 kg held in the participant's hand. The movement was triggered by the auditory stimulus synchronized with the start of the M-mode image registration. The frontal plane movement was chosen because previous research confirmed that images recorded during arm abduction presented fewer graphical distortions (Biały et al., 2017). Moreover, it has been reported that rotation forces in the direction of LAM fibers (especially TrA) are present during shoulder flexion/extension, which might have introduced movement-direction-dependent changes in the pattern of the LAM activation (Alison et al., 2008).

The optimal array location was marked on the skin with a piece of elastic kinesiotape when the US B-mode was on. Subsequently, the US device was switched to the M-mode, and participants took the 3-kg weight into their hand and performed three preparatory repetitions of the rapid arm abduction. The auditory stimulus appeared 2–6 s (time randomized) after participants proclaimed their readiness. One measurement included six repetitions of rapid arm abduction up to 90°, during which six M-mode US images were recorded on the opposite side of the trunk, as related to the moving arm. Then, after a 5-min rest interval, the contralateral LAMs were tested in the same manner. Additionally, the side of the body to be measured first was randomized. All the measurements were conducted in a laboratory setting under standardized environmental conditions. The reliability of the presented LAM measurement was reported earlier (Biały et al., 2017).

### Data Processing

For PA, the single static registration resulted in 300 sets of coordinates for all four markers (5 s × 60–Hz sampling frequency). The mean values of these 300 sets were calculated for *x, y*, and *z* coordinates. The virtual points representing the ASs and PSs were connected with four lines: AS R-L (joining right and left ASs), PS R-L (joining right and left PSs), AS-PS R (joining AS and PS on the right side), AS-PS L (joining AS and PS on the left side). The lines AS R-L and PS R-L were projected on the *xy* (frontal) plane, and the angles they formed with the reference *x*-axis (horizontal) were calculated: ∢FA (frontal anterior) and ∢FP (frontal posterior). In the sagittal plane, the AS-PS R and AS-PS L were projected on the *yz* plane, and the angles they formed with the reference *z*-axis (horizontal) were calculated: ∢SR (sagittal right), ∢SL (sagittal left). In the calculation protocol, positive values corresponded to the clockwise-oriented angle for the pelvis viewed from behind and from the right. Calculation of the frontal plane pelvic asymmetry index (PAIf) was based on the following formula: PAIf = (∢FA+∢FP)/2 (where: ∢FA: the angle between the AS R-L line and the horizontal; ∢FP: the angle between the PS R-L line and the horizontal). The zero value of the PAIf indicated perfect symmetry of the pelvis in the frontal plane (lines AS R-L and PS R-L were parallel to the *x*-axis, or the angles between them and *x*-axis were identical yet opposite). Positive values stood for lowering of the right innominate, negative values—for lowering of the left innominate. For the calculation of the sagittal plane pelvis asymmetry index (PAIs), the following formula was used: PAIs = ∢SR − ∢SL (where: ∢SR: the angle between the AS-PS R line and the horizontal; ∢SL: the angle between the AS-PS L line and the horizontal). The zero value of the PAIs indicated perfect symmetry of the pelvis in the sagittal plane (angles between lines AS-PS R / AS-PS L and *z*-axis were identical). Positive values stood for larger anterior rotation/flexion of the right innominate, while negative values for larger anterior rotation/flexion of the left innominate.

Considering LAM postural activity, twelve US images of the LAMs were gathered from each participant including six registrations for each side of the body. All images were evaluated for image quality by the third researcher, and two images of the lowest quality (e.g., due to graphical distortion) were excluded both from the right side image set as well as from the left side image set. A total of four images of the right LAMs and four images of the left LAMs were ultimately subjected to analysis. The images were transferred to the computer to be analyzed using Photoshop 8.0 (Adobe, San José, USA). The contrast (+75%) and zoom (× 10) of the images were adjusted. Then, using the Photoshop tools, the following measurements were taken for each individual LAM (TrA, internal oblique muscle (IO), and external oblique muscle (EO)): muscle thickness at rest, muscle thickness at the point of maximal activation (both along the *y*-axis of the image), time to achieve maximal activation (along the *x*-axis of the image). Both image axes were properly scaled to offer accurate information about time and spatial variables. Subsequently, the tissue deformation index (TDI) was calculated for each individual LAM according to the following formula: TDI = ((TA/TR×100%) − 100%) × T−1 (where: TR: muscle thickness at rest (mm); TA: muscle thickness at maximal activation (mm); T: time to achieve maximal activation (ms)). The mean TDIs from four images were subjected to statistical analysis.

### Statistical Analysis

Statistical analysis was performed using Statistica 10 software (StatSoft Inc., Tulsa, USA). The Shapiro-Wilk test was used to verify data distribution. Differences between PAIf and PAIs were assessed using the Mann-Whitney test. Kruskal-Wallis ANOVA was implemented to test differences between TDIs of the individual LAMs, with and without division into the right and left sides of the body. This test was followed by the Kruskal-Wallis ANOVA multiple comparisons post hoc test. The r-Spearmann test was used to evaluate correlations between different types of PA and TDIs. The alpha level was set at *p* = 0.05.

## Results

For the frontal plane PA mean PAIf of 1.44° was calculated. In 61 subjects, PAIf showed a positive value, which indicated right side asymmetry (lowering of right innominate bone). In 65 cases, left-side asymmetry (lowering of left innominate bone) was found. For the sagittal plane PA mean PAIs of 1.61° was calculated. In 57 cases, PAIs showed positive values, which indicated right side asymmetry (anterior tilt of right innominate bone), and in 69 cases left side asymmetry (anterior tilt of left innominate bone) was found (Table 1). No significant difference was observed between PAIf and PAIs magnitudes (Mann-Whitney test; Z = −1.04, *p* > 0.05). Moreover, no significant differences were revealed between right (PAIf or PAIs > 0) and left (PAIf or PAIs < 0) side asymmetries (for PAIf Z = −0.18, *p* > 0.05; for PAIs Z = 0.70 *p* > 0.05, respectively).

**Table 1:** Pelvic asymmetry indexes (PAIf and PAIs) divided into positive and negative values, the tissue deformation index (TDI) for individual lateral abdominal muscles with the respect to the body side.

Variables (*n* = 126)	*X*	−95%CI	+95%CI	min.	max.	SD
**PAIf (°)**	1.44	1.23	1.64	0.03	6.17	1.15
> 0	1.43	1.15	1.71	0.11	4.9	1.10
< 0	1.45	1.15	1.75	0.03	6.17	1.21
**PAIs (°)**	1.61	1.39	1.83	0.01	7.11	1.26
> 0	1.46	1.18	1.75	0.05	4.34	1.07
< 0	1.73	1.40	2.07	0.01	7.11	1,39
**TDI (%/ms)**						
TrA; R	0.047	0.071	0.057	0.001	0.26	0.051
TrA; L	0.070	0.084	0.057	0.001	0.25	0.063
IO; R	0.081	0.090	0.051	0.002	0.25	0.071
IO; L	0.087	0.095	0.049	0.003	0.26	0.078
EO; R	0.154	0.171	0.098	0.005	0.49	0.136
EO; L	0.150	0.168	0.103	0.004	0.49	0.133

PAIf, pelvic asymmetry index in the frontal plane; PAIs, pelvic asymmetry index in the sagittal plane; TDI, tissue deformation index; TrA, transversus abdominis; IO, internal oblique; EO, external oblique; R, right; L, left; > 0, positive values; < 0, negative values; X, mean; CI, confidence interval; min., minimal value; max., maximal value; SD, standard deviation

Differences of TDIs between the same LAMs (TrA, IO, and EO) located on the right and left sides of the body were statistically significant only in TrA (right TrA: 0.047 %/ms; left TrA: 0.070 %/ms; Mann-Whitney test: *Z* = 3.21, *p* < 0.01). The IO and EO inter-side differences were not significant (right IO: 0.081%/ms; left IO: 0.087%/ms; Mann-Whitney test: *Z* = 1.14, *p* > 0.05; right EO: 0.154%/ms; left EO: 0.149%/ms; Mann-Whitney test: *Z* = −0.41, *p* > 0.05) (Table 1).

In most cases, we found weak negative non-significant correlations between PAIf and TDIs, with only one exception of EO (PAIf-TrA right: *r* =−0.11, left: *r* =−0,06; PAIf-IO right: *r* =−0.15, left: *r* = −0.10; PAIf-EO right: *r* = 0.02, left: *r* = 0.12). On the contrary, analysis performed between PAIs and TDIs revealed weak positive non-significant correlations, with one exception of EO (PAIf-TRA right: *r* = 0.004, left: *r* = 0.003; PAIf-IO right: *r* = 0.05, left: *r* = 0.06; PAIf-EO right: *r* = 0.07, left: *r* = −0.02) (Table 2). We also performed an analysis of the correlation between the PA direction (left/right side PA) and TDIs. Considering correlations between PAIf (PAIf > 0 – right side asymmetry = lowering of right innominate bone; PAIf < 0 – left side asymmetry = lowering of left innominate bone) and TDIs, weak negative and non-significant correlations were found most often. The only exceptions were: PAIf > 0 − IO correlation (*r* = −0.35, *p* = 0.05) and PAIf < 0 − EO correlation (*r* = 0.29, *p* = 0.05). The same calculations were carried out for PAIs (PAIs > 0 – right side asymmetry = anterior tilt of right innominate bone; PAIs < 0 – left side asymmetry = anterior tilt of left innominate bone) and TDIs. For all tested variables, we recorded non-significant outcomes (all *p* > 0.05) (Table 2).

**Table 2:** Spearman *r* correlations between quantity of PA in the frontal and sagittal planes and LAM postural response with the respect to the body side.

Spearman *r* Correlations	Body side	TrA	IO	EO
**PAIf‒TDI**	R	−0.11	−0.15	0.02
	L	−0.06	−0.10	0.12
***p* level**		*p >* 0.05	*p >* 0.05	*p* > 0.05
**PAIs‒TDI**	R	0.004	0.05	0.07
	L	0.003	0.06	−0.02
***p* level**		*p* > 0.05	*p* > 0.05	*p* > 0.05

PAIf, pelvic asymmetry index in the frontal plane; PAIs, pelvic asymmetry index in the sagittal plane; TDI, tissue deformation index; TrA, transversus abdominis; IO, internal oblique; EO, external oblique; R, right; L, left; p > 0.05, not significant

## Discussion

This study is the first to investigate the relationship between two types of PA and LAM postural activity as expressed by PAIf/PAIs and TDIs, respectively. The main findings indicate that regardless of the type, magnitude, and direction of PA, there is no correlation with LAM TDI values without and with respect to the body side. There was no PA type or direction which could be regarded dominating in the study participants. For LAMs, we observed significant side-to-side differences only in TrA.

Based on our results, we can claim that pelvic spatial arrangement (expressed by PAIf and PAIs) is not correlated with LAM postural activity (expressed by TDI). Nevertheless, in the literature, data can be found indicating that PA is indirectly or directly related to the activity of LAMs. For example, Cibulka et al. (1988), after manual manipulation of the blocked SIJ of patients with lumbopelvic pain, registered a reduction in PA symptoms. Richardson et al. (2002) noticed a relationship between SIJ stiffness and LAM activity. Slightly different results, however, still supporting the relationship between LAMs, the SIJ and the pelvis, come from *in vitro* studies, where simulated TrA muscle activity reduced SIJ stiffness (Gnat et al., 2015). Also, Bashir et al. (2019) using US found reduced resting thickness of LAMs in patients with SIJ dysfunction. Similar conclusions were presented by Joseph et al. (2015). Parallel changes in PA and function of the pelvic girdle were also reported after therapeutic interventions on LAMs in the form of taping (Mehta et al., 2020) or exercise regimes engaging lower trunk musculature (Marzouk, 2019; Oleksy, 2019; Stuge et al., 2004). Despite the foundation in empirical data, our results cannot support the relationship between PA and postural activity of the LAMs. We found only very weak and rather non-significant correlations between them. In line with these outcomes, a lack of significant correlations between PA and LAMs was also shown by Polaczek and Szlachta (2023) in a group of 23 healthy children with diagnosed PA.

Apart from correlation analysis, which constituted the major objective of this study, it also can be concluded that PA is rather a common phenomenon. Morphology of the pelvic bones significantly determines the accuracy of PA measurements (Preece et al., 2008), and measurement error (for PAIf equal to 0.51°, and for PAIs equal to 0.82°) makes it impossible to record a fully symmetrical pelvis. At this point, the question should be asked: what value of the asymmetry index will indicate PA of clinical significance? Values below 1° are often found in healthy and physically active people (Ferreira et al., 2011). Therefore, it can be assumed that indexes below 1°, regardless of the type of asymmetry, are within the physiological range. Determining the cut-off point of 1° has also its statistical justification. For both PAIf and PAIs, the measurement error of the presented method is smaller than 1° (Gnat and Biały, 2015). Any result exceeding this value can, therefore, be regarded as an identifier of a true PA. It is also important that the PAIf was always accompanied by a smaller or a larger PAIs, which means that the two asymmetry types coexist with each other. Similar results were obtained in a pilot study in which reliability of the presented measurement method was evaluated. In 12 healthy people, Gnat and Biały (2015) recorded slightly different magnitudes of the PAIf and the PAIs along with a weak and non-significant correlation between them. In addition, the occurrence of right and left-side variants of PA was recorded with similar frequency. The right side variant occurred in the PAIf in 48.4%, while in the PAIs—in 45.2%. The magnitude of the right- and the left-side PA, both in the sagittal and frontal planes, also did not show any significant differences.

Results concerning LAMs only showed the characteristic TDI gradient (TrA < IO < EO), which has also been reported before (Biały et al., 2017) and could be explained by LAM morphology (Jourdan et al., 2020). We noticed significant side-to-side differences only in the TrA muscle TDI. The obtained results are, however, in opposition to opinions of other authors claiming that physiological LAM thickness is symmetrical (Rankin et al., 2006). Richardson et al. (2002) also reported symmetrical activation of LAMs, especially the TrA, during postural perturbation. On the contrary, Gray et al. (2016) found LAM thickness asymmetry in asymptomatic athletes, which can be complementary to our results, yet, in symptomatic subjects, they recorded LAM thickness symmetry. Kim et al. (2013) reported that TrA side-to-side thickness was characterized by the highest asymmetry as compared to other abdominal muscles, however, its thickness became symmetrical when a sudden, unexpected load acted on the torso. In turn, Adamczyk et al. (2015) analyzed changes in the thickness of LAMs during a rapid arm movement in a group of healthy basketball players and noted the highest magnitude of asymmetry in the TrA. Complementary results were found in runners (Mitchell et al., 2019). Based on these claims, it can be concluded that TrA activity is rather asymmetrical ant that this asymmetry does not necessarily indicate the presence of any pathology. Our results also revealed TrA asymmetry and, in the author's opinion, this asymmetry can be considered a physiological feature of this particular muscle.

The main limitation of the study is the profile of our research group, which, despite its relative homogeneity, consisted of relatively young subjects with no dysfunction or pain within the lumbopelvic region. Therefore, attention must be paid during any generalizations, and interpretations should not go far beyond the limits set by the group's profile. In PA measurement, the precise palpation of the anterior and posterior superior iliac spines is a crucial factor for obtaining an adequate level of measurement reliability, which may be jeopardized by an excessive amount of fat tissue. The limitation of LAM US measurement refers to the position and pressure of the US array during postural perturbation in the form of a rapid arm movement. We attempted to effectively minimize it by adequate training of the raters and thorough standardization of the measurement procedure.

## Conclusions

No evidence was found to support the thesis that PA is related to LAM postural activity. Regardless of the type, magnitude, and direction of PA, there is no correlation between PA and the LAM TDI, without and with respect to the body side. Moreover, in the group of young, healthy, and relatively active people, two types of PA coexist, although none of them predominates, and each occurs with a similar frequency in the right- or the left-side variant. The TrA muscle TDI shows significant side-to-side asymmetry, which does not occur in more superficial IO and EO muscles. This information may be used as a reference point for clinical populations with various lumbopelvic dysfunctions.
